# The impact of race on postpartum opioid prescribing practices: a retrospective cohort study

**DOI:** 10.1186/s12884-021-03954-8

**Published:** 2021-06-22

**Authors:** Tyler R. McKinnish, Adam K. Lewkowitz, Ebony B. Carter, Ashley E. Veade

**Affiliations:** 1 Department of Obstetrics and Gynecology, Washington University in St. Louis, 4901 Forest Park Ave, St. Louis, MO 63108 USA; 2 Woman and Infants Hospital of Rhode Island, Alpert Medical School of Brown University, Providence, RI USA

**Keywords:** Race, Racial disparities, Health disparities, Opioids, Narcotics, Postpartum pain

## Abstract

**Background:**

To identify the association between inpatient postpartum opioid consumption, race, and amount of opioids prescribed at discharge after vaginal or cesarean delivery.

**Methods:**

A total of 416 women who were prescribed an oral opioid following vaginal or cesarean delivery at a single tertiary academic institution between July 2018 and October 2018 were identified. Women with postoperative wound complications, third and fourth degree lacerations, cesarean hysterectomy, or a history of opioid abuse were excluded. The primary outcome was the number of oxycodone 5 mg tablets prescribed at discharge, stratified by race and mode of delivery. Only “Black” and “White” women were included in analyses due to low absolute numbers of other identities. Black women were compared to white women using multivariable logistic regression. Multiple sensitivity analyses were performed.

**Results:**

The median number of oxycodone tablets consumed during hospitalization following cesarean delivery was seven (IQR: 2.5–12 tablets) and following vaginal delivery was one (IQR: 0–3). White women were more likely to be older at delivery regardless of route (median 32 vs. 30 years for cesarean delivery, and 29 vs. 27 years for vaginal delivery; *p *< 0.01 for both). White women undergoing cesarean delivery did so at a lower maternal BMI (31.6 vs. 34.5; *p* = 0.02). White women were also significantly more likely to have private insurance and to experience perineal lacerations following vaginal delivery. The number of inpatient opioid tablets consumed, as well as the number prescribed at discharge, were not statistically different between Black and White women, regardless of mode of delivery. These findings persisted in sensitivity analyses.

**Conclusion:**

At our large, academic hospital the number of tablets prescribed at discharge had no association with patient race or inpatient usage regardless of mode of delivery.

## Background

According to the Centers for Disease Control and Prevention (CDC), over 63,000 people died from drug overdose in 2016 [1]. Of these deaths, 42,251 (66%) involved opioids overdoses. In the following year, this number continued to climb with 47,600 opioid overdoses in 2017 [2]. The role of the healthcare system in the perpetuation of this epidemic is clear, as 40% of opioid overdose related deaths are attributable to prescription narcotics [3]. Opioids are commonly used in the management of postpartum pain, making obstetrics practitioners important stakeholders in this ongoing public health crisis.

In one large study of privately insured patients who had undergone vaginal delivery, 28.5% filled an opioid prescription for a median of 20 tablets of 5 mg (mg) oxycodone within six weeks of delivery [4]. Of patients undergoing cesarean section, approximately 85% filled a prescription for a median of 40 tablets despite consuming a median of only 20 tablets [5]. Overprescribing opioids is not without risk: 1.7% of patients who underwent vaginal delivery and 2.2% of cesarean deliveries had new persistent opioid use (> 90 days after delivery) following initiation at delivery [6].

Numerous studies have shown significant racial and ethnic disparities in opioid prescribing practices. A Prescription Drug Monitoring Program (PDMP) study in California found 300% higher prescription rates in predominantly White communities compared to non-White communities [7]. Furthermore, though Black and Hispanic women report higher pain scores in the postpartum period, they receive fewer inpatient morphine milligram equivalents (MMEs) compared to White women [8]. This difference was statistically significant, though of uncertain clinical significance given the very small absolute difference in MME [9]. Badreldin et. al (2019) also found that Black women are less likely to receive a prescription for opioids on discharge. To our knowledge, no other study has yet examined whether racial differences in inpatient prescriptions translate to differences in opioids prescribed at discharge. As such, this study aims to investigate the effect of race on inpatient postpartum opioid consumption and the amount of opioids prescribed at discharge after vaginal or cesarean delivery.

## Methods

This retrospective cohort study identified women undergoing vaginal and cesarean delivery at a single tertiary academic institution in the Midwest between July 2018 and October 2018 who had an active oral opioid order as an inpatient within the electronic medical record. The electronic medical record (EMR) was reviewed to identify eligible women. Sociodemographic and clinical information, including age, BMI measured on admission, insurance status, and self-reported race, was extracted from the records of qualifying subjects. This study was performed in accordance with the Declaration of Helsinki and was approved by The Washington University in St. Louis Human Research Protection Office (IRB # 201,901,055).

Patients were excluded if they had postoperative wound complications (during admission or after discharge), third and fourth degree perineal lacerations, cesarean hysterectomy, or a known history of antenatal opioid use, as these were thought to potentially increase post-partum opioid usage, influence prescriber behavior, and bias or confound results. Antenatal opioid abuse was defined as a known diagnosis of opioid abuse or receipt of greater than one opioid prescription (including buprenorphine and methadone) during pregnancy as identified by comprehensive review of the electronic medical record. Wound complications were defined as wound infections, seromas, or hematomas requiring surgical or medical intervention.

Inpatient usage and amount of narcotics provided at discharge were identified. Oxycodone 5 mg is the preferred postpartum narcotic for all providers at our institution, comprising 95% of discharge opioid prescriptions, with Tramadol 50 mg, oxycodone 5 mg-acetaminophen 325 mg, hydrocodone 5 mg-acetaminophen 325 mg, and hydrocodone 7.5 mg-acetaminophen 325 mg combination tablets comprising the remaining 5%. The few forms of oral opioids that were not oxycodone tablets were converted to morphine milligram equivalents (MMEs) and then converted to oxycodone 5 mg tablets for ease of analysis and comparison. Parenteral opioid administration following delivery is exceedingly rare at our institution and was not calculated as part of this analysis, as it was thought to be reflected in higher oral opioid usage. All patients had maximum safe dosages of acetaminophen and ibuprofen available as first line analgesics, except where contraindicated.

The primary outcome was the number of oxycodone 5 mg tablets prescribed at discharge, stratified by race. Race was categorized as “Black” and “White,” with all other racial or ethnic identities excluded due to low absolute numbers of each. Sociodemographic data abstracted from the medical record included age, race, body mass index (BMI), insurance payer, length of post-delivery stay in days, and the discharge opioid prescriber type (intern, resident, or attending).

Patients were stratified by mode of delivery. Demographic and baseline clinical data were compared between Black and White women using a chi-squared test or Fisher’s exact test for categorical variables, as appropriate, and the Student’s t-test or Mann–Whitney U-test for continuous variables. Sensitivity analyses were then performed for those who underwent vaginal delivery, excluding women who underwent bilateral tubal ligation (BTL), and including only women who received no postoperative oxycodone tablets as inpatients despite having an order for narcotics. Sensitivity analysis was also performed excluding women who underwent cesarean delivery with hospital length of stay > 5 days in order to exclude possible effects of serious intraoperative or postoperative complications and other health conditions, and including only women who received no postoperative oxycodone tablets as inpatients despite having an order for narcotics. Multivariable logistic regression was used to adjust for type of laceration, insurance status, age, and BMI for vaginal deliveries, and age and BMI for cesarean deliveries. No adjustments were not made for group sizes smaller than *n* ≤ 5 to avoid overfitting of the model.

## Results

A total of 416 patients were included in this analysis, 268 of whom underwent cesarean delivery and 148 of whom underwent vaginal delivery.

### Cesarean delivery

**Fig. 1:**
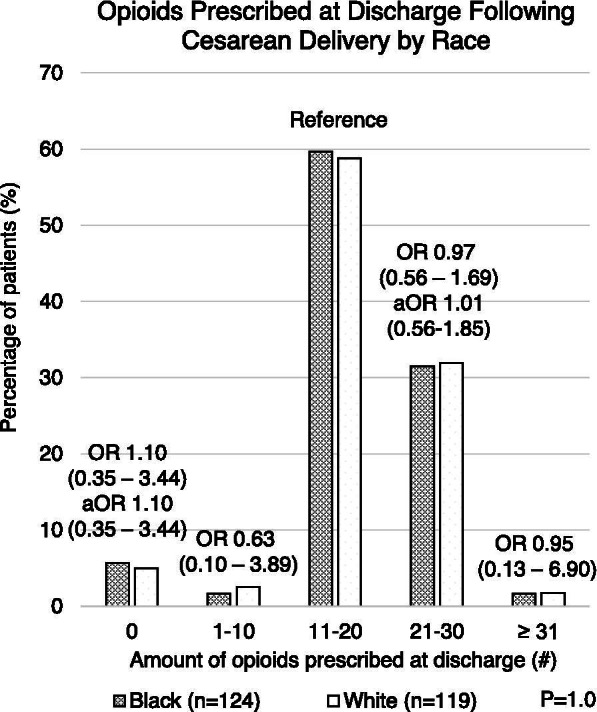
Number of oxycodone 5 mg tablets prescribed at discharge following cesarean delivery by race. Multivariable logistic regression was used to adjust for age and BMI in groups with *n* > 5

Among the 268 women who underwent cesarean delivery, the median number of oxycodone tablets consumed while inpatient following delivery was seven (interquartile range (IQR) 2.5–12 tablets). Slightly more than half (51.0%) of delivering women identified as Black. White women were more likely to deliver at higher maternal age (median 32 vs. 30 years; *p *< 0.01) and with lower maternal BMI (31.6 kg/m^2^ vs. 34.5 kg/m^2^; *p* = 0.02) compared to Black women (Table 1). White women were also significantly more likely to have private insurance (*p *< 0.01). There was no significant difference between Black and White women in the total number of inpatient opioid tablets consumed (*p* = 0.074) (Table 1). There was no association between number of opioid tablets prescribed at discharge and patient race in unadjusted analyses, or when adjusted for age and BMI (0 tablets aOR 1.1 [0.35–3.44], 1–10 tablets OR 0.63 [0.10–3.89] unadjusted due to small group sizes, 11–20 tablets [Reference], 21–30 tablets aOR 1.01 [0.56–1.85], and ≥ 31 tablets OR 0.95 [0.13–6.90] unadjusted due to small group sizes; *p* = 1.0) (Fig. 1). Women who consumed zero tablets inpatient (*n* = 38) received a median of 20 oxycodone 5 mg tablets (IQR 0–40) at time of discharge with no difference between patient race (Table 2). These results did not differ in a separate sensitivity analysis in which patients with prolonged recovery necessitating discharge after postpartum day five were excluded (Table 3).

**Table 1:** Demographic factors and inpatient opioid usage for all patients prescribed narcotics following cesarean delivery stratified by race

Characteristic	Black (*n* = 124)	White (*n* = 119)	*P* value
**Age at delivery** **(median (IQR))**	30 (26–33)	32 (28–35)	0.0001*
**Insurance Type**			
Private Insurance	49%	85%	< 0.0001*
Public Insurance	48%	15%	
Other	2%	0%	
**Maternal BMI** **(median (IQR))**	34.5 (27.9–41.0)	31.6 (26.2–37.5)	0.02*
**Day of discharge** **(n (%))**			
PPD 3 or less	46%	45%	0.12
PPD 4	38%	50%	
PPD 5 or more	10%	5%	
**Number of inpatient tablets (n (%))**			
0	11%	20%	0.074
1–6	31%	34%	
7–12	29%	29%	
13 +	29%	17%	
**Discharge opioid prescribing provider (n (%))**			
Intern	61%	55%	0.6
Resident	28%	30%	
Attending	11%	15%	

^*^ Statistical significance (*p *< 0.05)

**Table 2:** Sensitivity analysis including only patients who consumed zero inpatient opioids following cesarean delivery

# Oxycodone 5 mg tablets prescribed at discharge (n (%))	Black (*n* = 14)	White (*n* = 24)	Unadjusted OR	*P *value
0	2 (14.3)	2 (8.3)	1.60 (0.19–13.24)	0.7
1–10	1 (7.1)	1 (4.2)	1.60 (0.090–28.57)	
11–20	10 (71.4)	16 (66.7)	Reference	
21–30	1 (7.1)	5 (20.8)	0.32 (0.32–3.15)	
≥ 31	0 (0.0)	0 (0.0)	–	

^*^ Statistical significance (*p *< 0.05)

**Table 3:** Sensitivity analysis excluding patients discharged after postoperative day five

# Oxycodone 5 mg tablets prescribed at discharge (n (%))	Black (*n* = 112)	White (*n* = 113)	Unadjusted OR	*P* value	Adjusted OR**
0	7 (6.3)	6 (5.3)	1.20 (0.38 – 3.77	1.0	1.27 (0.33 – 4.87
1–10	2 (1.8)	3 (2.7)	0.69 (.11 – 4.25)		–
11–20	64 (57.1)	66 (58.4)	Reference		Reference
21–30	37 (33.0)	37 (32.7)	1.03 (0.58 – 1.83)		1.09 (0.58 – 2.05)
≥ 31	2 (1.8)	1 (0.9)	2.07 (0.18 – 23.31)		–

^*^ Statistical significance (*p *< 0.05) **Adjusted for age and BMI

### Vaginal delivery

**Fig. 2:**
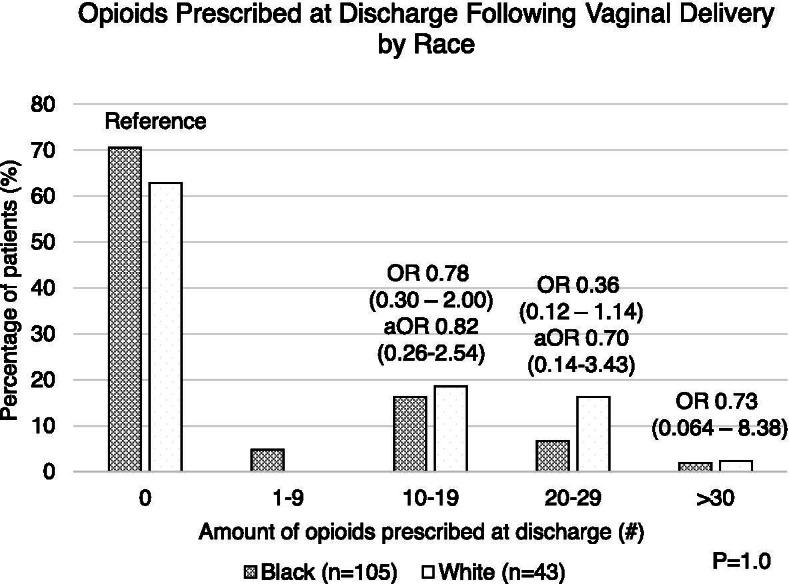
Number of oxycodone 5 mg tablets prescribed at discharge following vaginal delivery (with and without BTL) by race. Multivariable logistic regression was used to adjust for type of laceration, age, insurance status, and BMI in groups with *n* > 5

Among 148 women, the median number of oxycodone tablets consumed while inpatient following delivery was one (IQR 0–3 tablets). The majority (70.9%) of delivering women identified as Black. White women were more likely to deliver at higher maternal age (median 29 vs 27 years; *p* = 0.008), to have private insurance (*p *< 0.0001), and to experience perineal lacerations following delivery (*p *= 0.005) compared to Black women (Table 4). The number of inpatient oxycodone tablets consumed by Black and White women was not statistically different (*p *= 0.7) (Table 4). There was no association between race and number of tablets prescribed at discharge among all women following vaginal delivery in unadjusted analyses, or when adjusted for obstetric laceration, insurance status, age, and BMI (0 tablets [Reference], 1–9 tablets [Null], 10–19 tablets aOR 0.82 [0.26–2.54], 20–29 tablets aOR 0.70 [0.14–3.43], and ≥ 30 tablets aOR 1.23 [0.052–29.02]) (Fig. 2). These findings persisted in separate sensitivity analyses including only patients who utilized zero tablets inpatient (*p *= 0.3) (Table 5), and excluding patients who underwent a postpartum BTL (*p *= 0.3) (Table 6).

**Table 4:** Demographic factors and inpatient opioid usage for all patients prescribed narcotics following vaginal delivery stratified by race

Characteristic	Black (*n* = 105)	White (*n* = 43)	*P* value
**Maternal age at delivery (median (IQR)) years**	27 (22–31)	29 (23–33)	0.008*
**Insurance Type (n (%))**			
Private Insurance	14 (13.3)	28 (65.1)	< 0.0001*
Public Insurance	88 (83.8)	14 (34.9)	
Other	3 (2.9)	0 (0.0)	
**Maternal BMI (median (IQR))**	32.1 (26.6–36.9)	29.3 (25.5–35.7)	0.2
**Type of delivery (n(%))**			
Spontaneous	96 (91.4)	40 (93.0)	0.8
Vacuum assisted	5 (4.8)	1 (2.3)	
Forceps assisted	4 (3.8)	2 (4.7)	
**Type of laceration (n (%))**			
Intact	66 (64.1)	14 (32.6)	0.005*
1^st^ degree	12 (11.6)	5 (11.6)	
2^nd^ degree	11 (10.7)	14 (32.6)	
3^rd^ degree	1 (1.0)	1 (2.3)	
4^th^ degree	1 (1.0)	0 (0.0)	
Other (peri-urethral, sulcal)	12 (11.6)	9 (20.9)	
**Complications (n (%))**			
None	88 (83.8)	35 (81.4)	0.4
BTL	14 (13.3)	4 (9.3)	
Cervical laceration	1 (1.0)	1 (2.3)	
Other (Episiotomy, IUFD, DVT, trauma)	2 (1.9)	3 (7.0)	
**Number of inpatient tablets consumed (n (%))**			
0	25 (23.8)	13 (30.2)	0.7
1	28 (26.7)	8 (18.6)	
2	13 (12.4)	5 (11.6)	
3 or more	39 (37.1)	17 (39.5)	
**Discharge opioid prescribing provider (n (%))**			
Intern	39 (37.1)	18 (41.9)	0.8
Resident	52 (49.5)	19 (44.2)	
Attending	14 (13.3)	6 (13.9)	

^*^ Statistical significance (*p *< 0.05)

**Table 5:** Sensitivity analysis excluding patients who consumed zero inpatient opioids following vaginal delivery (with or without BTL)

# Oxycodone 5 mg tablets prescribed at discharge (n (%))	Black (*n* = 25)	White (*n* = 13)	Unadjusted OR	*P* value
0	16 (64.0)	9 (69.2)	Reference	0.3
1–9	4 (16.0)	0 (0.0)	–	
10–19	4 (16.0)	2 (15.4)	1.13 (0.17–7.40)	
20–29	1 (4.0)	2 (15.4)	0.28 (0.022–3.55)	
≥ 30	0 (0.0)	0 (0.0)	–	

^*^ Statistical significance (*p *< 0.05)

**Table 6:** Sensitivity analysis excluding patients who underwent BTL following vaginal delivery

# Oxycodone 5 mg tablets prescribed at discharge (n (%))	Black (*n* = 91)	White (*n* = 39)	Unadjusted OR	Adjusted OR**
0	71 (78.0)	26 (66.7)	Reference	Reference
1–9	4 (4.4)	0 (0.0)	–	–
10–19	9 (9.9)	7 (18.0)	0.47 (0.16–1.39)	0.39 (0.10–1.50)
20–29	5 (5.5)	6 (15.4)	0.31 (0.86–1.09)	1.08 (0.18–5.83)
≥ 30	2 (2.2)	0 (0.0)	–	–

^*^ Statistical significance (*p *< 0.05) **Adjusted by laceration, insurance type, age, and BMI

## Discussion

In this study, we detected no difference in inpatient postpartum opioid use by race. Despite statistically significant differences in age, insurance status, perineal lacerations, and BMI at time of delivery, inpatient postpartum use of oxycodone 5 mg tablets was nearly identical between Black and White patients. This is in contrast to the findings of other institutions, which note significant differences in opioid prescription practices both in the postpartum period and in numerous other specialties and situations between Black and White patients [8, 10, 11]. In addition, we found that the amount of opioid tablets prescribed at discharge were not associated with patient race. These findings persisted even in patients who did not request or receive inpatient opioids for pain control despite being able to receive them. To our knowledge, this is the first study to compare opioid prescriptions at discharge stratified by patient race and inpatient usage.

There are multiple possible explanations for why there was a lack of association identified between patient race and opioids prescribed at discharge in this study [8, 12]. First, it may reflect a difference in patient population, as the study by Badreldin et al. included over 9900 patients but only 10.6% of whom identified as non-Hispanic Black, whereas 66% of women in our study population identified as Black. In a similar study by Johnson et al., 25% of patients identified as non-Hispanic Black and received fewer opioid doses in the immediate postpartum period compared to their White counterparts, despite higher pain scores [12]. These studies reported on primarily White populations, and neither reported on the amount of opioids provided at discharge, which in this study made up the bulk of opioids prescribed. Another possible explanation is that at our academic institution, intern or resident physicians (of which there are 36; 77% female, 83% White) prescribe the vast majority of discharge opioids. All residents train and provide care in both outpatient and inpatient environments which serve a predominantly Black patient population and receive formal training on techniques to reduce implicit bias, which may have effectively decreased racial disparities in opioid prescription. Lastly, our primary findings may also simply reflect that at the time of this study, there were no specific institutional guidelines for discharge opioid prescriptions. In the absence of guidelines, residents prescribe varying, largely arbitrary numbers of tablets from 0–30, based on the resident’s discretion. This may have translated into a lack of racial or ethnic inequity in opioid prescribing, though may also be insensitive to cultural differences in the temporal experience of pain and risks over- or under-prescribing for some patients.

This study is strengthened by its inclusion of all routine vaginal and cesarean deliveries with an inpatient postpartum opioid order from six different obstetric practices with a diverse payer mix at a large institution for the analyzed period. Obstetric care providers at our institution order opioids for all patients who have undergone cesarean delivery without a contraindication to opioids, as well as patients who have undergone vaginal delivery with BTL or without BTL if they experience moderate-severe or persistent pain, when non-narcotic analgesics fail or are contraindicated, or per patient request, making this a diverse sample of patients experiencing postpartum pain. The medical records of all patients included in the study were also reviewed and abstracted by obstetricians, adding validity to our findings.

This study is not without limitations, particularly its small sample size in the setting of overall decreased postpartum opioid prescribing. We also did not incorporate postpartum pain scores as, though these values are collected by bedside nurses, obstetric providers do not reliably utilize these metrics during throughout admission or review them at the time of patient discharge. Instead, patient requests for additional analgesia and safe dosing interval- not an arbitrary pain threshold- are the determining factors for initial opioid administration and subsequent frequency. Nevertheless, pain scores may have added a dimension of quantification to the outcome of our study, perhaps identifying patients with undertreated pain despite receiving equivalent doses of opioids. It is important to note that pain scores do not account for patient preference or beliefs about opioid analgesia and goals for pain management, and they may be insensitive to cultural differences in the perception or reporting of pain, so may also be misleading [13].

In summary, this study contributes an important addition to the literature regarding racial disparities in postpartum practices. In our diverse patient population, we did not note significant differences in inpatient postpartum opioid usage or discharge prescribing practices by patient race. In light of recent nationwide, professional, and institutional efforts to stem the tide of opioid use disorder and opioid related deaths, this study provides a baseline for our institution and others like it by which to measure the racial equity of these interventions. As postpartum opioid prescribing practices change, it will be important to be mindful of implicit and explicit biases and to specifically study interventions in predominantly minority populations to prevent these changes from unequally affecting minority women.

## Conclusions

In this retrospective cohort study of postpartum patients at a large tertiary academic institution with a primarily Black patient population, there was no difference in either inpatient opioid use or amount prescribed at discharge by race. This study provides an important baseline for institutions like ours by which to measure the equity of the impacts of strategies to quell the opioid epidemic. Prescribers should continue to survey prescription data at their institutions to ensure that protocolized prescribing and conservative opioid distribution do not unequally affect minority patients.

## Data Availability

The datasets used and/or analyzed during the current study are available from the corresponding author on reasonable request.

## Abbreviations

CDC: Centers for Disease Control and Prevention
PDMP: Prescription drug monitoring program
MME: Morphine milligram equivalents
mg: Milligrams
EMR: Electronic medical record
BMI: Body mass index
BTL: Bilateral salpingectomy or tubal ligation
OR: Odds ratio
aOR: Adjusted odds ratio
IQR: Interquartile range
